# Failure of diltiazem to prevent 1:1 conduction of atrial flutter: a case report

**DOI:** 10.1186/s13256-023-03947-5

**Published:** 2023-07-18

**Authors:** K. D. Tiver, D. K. Martin, J. Quah, A. Lahiri, A. N. Ganesan

**Affiliations:** 1 Department of Cardiology, Flinders Medical Centre, Level 6, Flinders Drive, Bedford Park, SA 5042 Australia; 2 College of Medicine and Public Health, Flinders University, Bedford Park, SA 5042 Australia; 3 Department of Orthopaedic Surgery, Flinders Medical Centre, Bedford Park, SA 5042 Australia

**Keywords:** Atrial flutter, 1:1 conduction, Flecainide, Diltiazem, Class 1c, Case report

## Abstract

**Background:**

Atrial flutter with 1:1 conduction to the ventricles is a dangerous cardiac arrhythmia. Contemporary guidelines recommend atrioventricular nodal blocking agents should be co-administered with class 1C anti-arrhythmics, as prophylaxis against 1:1 flutter. No guidance is provided on the type or strength of atrioventricular nodal blockade required, and in practice, these agents are frequently prescribed at low dose, or even omitted, due to their side effect profile.

**Case presentation:**

A 62 year old Caucasian man with a history of paroxysmal atrial fibrillation treated with flecainide, presented with atrial flutter with 1:1 conduction to the ventricles and was cardioverted. Diltiazem was added to prevent this complication and he again presented with atrial flutter with 1:1 conduction to the ventricles, despite prophylaxis with coadministration of diltiazem.

**Conclusions:**

This case report demonstrates failure of diltiazem to prevent 1:1 flutter in a patient chronically treated with flecainide for paroxysmal atrial fibrillation.

## Background

Atrial fibrillation (AF) is a common problem, with a lifetime risk of 1 in 4 of developing the condition [1]. It was estimated in 2016, that 46.3 million individuals worldwide have AF, and AF prevalence is thought to have increased threefold over the last 50 years [2]. AF is a risk factor for stroke, and it is associated with increased mortality, as well as a range of complications, including heart failure, myocardial infarction, chronic kidney disease, venous thromboembolism and dementia [2].

Class 1c antiarrhythmics are commonly prescribed, as they are effective in maintaining sinus rhythm [3]. This class of medications is reported to cause 1:1 atrial flutter in 3.5–5% of patients chronically treated [4], due to slowing of atrial rate. This can potentially be a life threatening complication, if the rhythm degenerates into ventricular fibrillation. Flecainide, a class 1c antiarrhythmic, has been reported to convert atrial fibrillation to atrial flutter with 1:1 conduction to the ventricles [5], and atrioventricular (AV) nodal blocking agents are said to offer some protection from this event [6]. As such, current guidelines recommend the co-prescription of AV nodal blocking agents together with class 1c agents to prevent this complication [7–9]. Despite this being a unanimous recommendation, there is no current evidence or guideline on the type or dose of AV nodal blocking agent needed to reduce this risk.

## Case presentation

A 62 year old Caucasian medical professional, with reliable compliance, presented to the cardiology clinic with a two hour history of palpitations, chest tightness and presyncope. This occurred on the background of a 15 year history of paroxysmal atrial fibrillation, treated with flecainide, orally, 100 mg twice daily, without concomitant use of an AV nodal blocking agent at the time of presentation. He had no previous invasive procedures for atrial fibrillation, and flecainide was the first line antiarrhythmic agent used in this man. There were no clinical risk factors for atrial fibrillation, including no history of hypertension, obstructive sleep apnoea, alcohol overuse, diabetes or heart failure. There was no family history of atrial fibrillation or cardiac disease. His CHA_2_DS_2_-VASc score was 0, and therefore he was not anticoagulated. He experienced similar symptoms 4 days prior, which spontaneously resolved after 3 h.

**Fig. 1:**
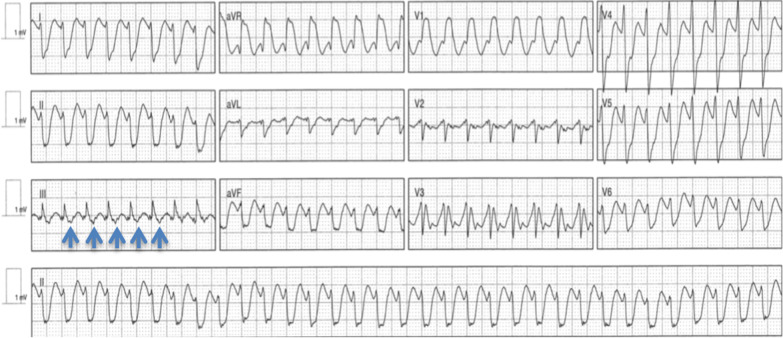
12 lead electrocardiogram at presentation, demonstrating atrial flutter with 1:1 atrioventricular conduction. Arrows in lead III mark the flutter waves, demonstrating negative flutter waves in this lead, consistent with typical cavotricuspid isthmus (CTI) flutter, a counterclockwise macro re-entrant circuit in the right atrium. Due to aberrant conduction, the axis of this electrocardiogram is “north-west” or “extreme axis deviation” and there is an atypical right bundle branch block appearance

**Fig. 2:**
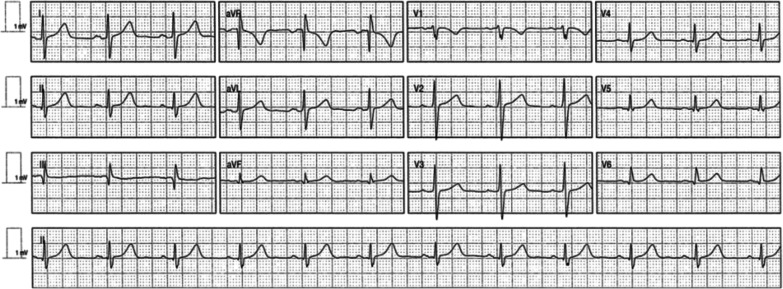
12 lead electrocardiogram in sinus rhythm. Normal sinus P waves, normal PR interval, normal QRS duration, normal QRS axis, normal QT interval, no T wave changes

His 12 lead electrocardiogram (ECG) showed atrial flutter with 1:1 conduction, at 200 beats per minute, with a morphology consistent with typical cavotricuspid isthmus (CTI) flutter (Fig. 1). Systolic blood pressure was 90 mmHg. Intravenous metoprolol was initially trialled, but caused hypotension, so he proceeded to receive direct current cardioversion, which successfully reverted him to sinus rhythm. His sinus rhythm ECG is shown in Fig. 2. His echocardiogram was normal. He was discharged on apixaban 5 mg tablet twice daily and diltiazem 180 mg controlled release daily, with plans for elective CTI ablation. The diltiazem dose was chosen with consideration of bradycardia (heart rate 45 bpm) and hypotension (BP 100/60) at the time of discharge.

He represented to the cardiac clinic 5 days later with recurrent palpitations and chest tightness after climbing a flight of stairs. Repeat ECG again confirmed atrial flutter with 1:1 conduction, and systolic BP was 80 mmHg. Direct current cardioversion was successful and he underwent successful radiofrequency CTI catheter ablation. He was discharged on flecainide and has had no further recurrence of atrial flutter or fibrillation.

Ongoing therapy with apixaban, flecainide and diltiazem was well tolerated, and the patient, a reliable medical professional, reported full adherence to therapy.

The patient reported he had coped with episodes of atrial fibrillation for many years, but he felt much more unwell with the episode of atrial flutter, requiring cardioversion in the first instance. He was glad to be placed on a medication which he was assured would prevent further episodes of 1:1 atrial flutter, which would allow him to continue with his activities of daily living. He was somewhat dismayed that having been on diltiazem for 5 days, he went back into 1:1 flutter whilst climbing a flight of stairs at the hospital, necessitating a further cardioversion and subsequent atrial flutter ablation procedure.

## Discussion

Atrial flutter with 1:1 AV nodal conduction can be associated with haemodynamic compromise and adverse clinical outcomes. Flecainide can predispose to 1:1 conduction of atrial flutter as it slows the intra-atrial as well as the atrio-ventricular conduction. Current international guidelines [7–9] recommend an AV nodal blocking agent to be co-prescribed with flecainide, in order to prevent 1:1 AV nodal conduction in atrial flutter. Despite this being recommended, limited guidance is provided as to implementation of this recommendation in practice. Specifically, there is no guidance on the type or dose of AV nodal agent required to prevent 1:1 flutter, and these medications are often prescribed at low dose, given the side effect profile of these agents. In particular in clinical practice, these agents cause bradycardia, hypotension and lethargy, which limits their use.

There are case reports of failure of atenolol, digoxin and verapamil to prevent 1:1 atrial flutter [10], however to our knowledge there are no such reports with diltiazem. There is limited awareness in the prescribing community that non-dihydropyridine calcium channel blockers (verapamil, diltiazem) can be ineffective at preventing 1:1 flutter, when co-administered with flecainide. This case report highlights an example of recurrent 1:1 atrial flutter despite diltiazem, and a gap in the evidence base to guide clinical practice.

Several groups have looked into the predictors of 1:1 atrial flutter; perhaps this information may be useful in the decision-making around dose and type of AV nodal blockade required. The recurrent atrial flutter with 1:1 conduction in this case occurred with exertion, in this case walking up a flight of stairs. This is theoretically more likely, due to increased sympathetic tone, and a previous case series of 8 patients with 1:1 atrial flutter reported that all cases occurred with exertion [10]. It should be noted however, that there has been a recent report of atrial flutter with 1:1 conduction occurring even at rest after flecainide administration [11]. Others have noted that a short PR interval, indicating rapid potential AV nodal conduction, and a longer atrial flutter cycle length are predisposing factors to atrial flutter with 1:1 conduction in patients treated with class 1 antiarrhythmic medications.

In terms of the choice of anti-arrhythmic therapy in this patient, he had been on long term flecainide therapy with good efficacy in prophylaxis against symptomatic AF episodes. He developed a separate but related arrhythmia, typical CTI dependent atrial flutter, which can co-exist and interact with AF. In this instance, the strategy used was to eliminate the CTI dependent atrial flutter, with radiofrequency ablation, and continue the flecainide for AF. The concomitant use of an AV nodal blocking agent was designed to prevent rapidly conducted atrial flutter in the case of atrial flutter recurrence.

This case illustrates an example of diltiazem 180 mg not providing enough AV nodal blockade to prevent 1:1 flutter with exertion in this patient on flecainide therapy; but it is unclear if this would have been sufficient for the same patient on a lower dose of flecainide, or whether equivalent dose beta blocker may have prevented 1:1 atrial flutter. We don’t fully understand whether there is a role for using supplemental information that may indicate higher risk of 1:1 flutter, such as baseline markers of AV nodal conduction (PR interval) or the atrial flutter cycle length, to guide the decision for dose and type of AV nodal blockade required.

## Conclusions

Guidelines recommend that AV nodal blocking agents should be co-administered with flecainide in order to prevent atrial flutter with 1:1 conduction to the ventricles. We present a case of recurrent 1:1 atrial flutter despite guideline based diltiazem therapy. There is limited awareness in the prescribing community of failure of diltiazem to prevent 1:1 atrial flutter, and in real-world practice, AV nodal blocking agents are often given at very low dose owing to their side effect profile.

## Data Availability

Data sharing not applicable to this article as no datasets were generated or analysed during the current study.

## Abbreviations

AV: Atrioventricular
ECG: Electrocardiogram
CTI: Cavotricuspid isthmus
AF: Atrial fibrillation
